# Equitable recovery from COVID-19: bring global commitments to community level

**DOI:** 10.1136/bmjgh-2020-004757

**Published:** 2021-01-17

**Authors:** Rene Loewenson, Lucia D'Ambruoso, Duong Minh Duc, Reidar Hjermann, Winfred Lichuma, Elizabeth Mason, Elizabeth Nixon, Norma Rudolph, Eugenio Villar

**Affiliations:** 1 Training and Research Support Centre, Harare, Zimbabwe; 2 Institute of Applied Health Sciences, University of Aberdeen School of Medicine, Medical Sciences and Nurition, Aberdeen, UK; 3 Department of Population and Reproductive Health, Faculty of Social & Behavioural Sciences, Hanoi University of Public Health, Hanoi, Viet Nam; 4 Norwegian Psychology Association, Oslo, Norway; 5 Osimbo Lichuma Advocates, Nairobi, Kenya; 6 Department of Infectious Disease Epidemiology, London School of Hygiene & Tropical Medicine, London, UK; 7 School of Psychology, Trinity College Dublin, Dublin, Ireland; 8 Independent researcher, Tampere, Finland; 9 Facultad de salud pública y administración, Universidad Peruana Cayetano Heredia, Lima, Peru

**Keywords:** COVID-19, public health, health policy

**Summary box**

- High level speakers at the December 2020 United Nations General Assembly pointed to the growing inequalities and stress to health, social, economic and democratic systems caused by COVID-19, calling for a range of collective interest driven responses and measures for a sustainable recovery.
- The pandemic, lockdown and other responses, along with underfunded, poorly prepared and overstretched public sector social and health systems in many countries worsened many dimensions of family, women’s, child and adolescent health and well-being that were already facing deficits, generating a rising health and social debt in communities, the true scale and long-term consequences of which are as yet unknown, especially for the most marginalised in society.
- Rather than ‘getting back to normal’, recovery and ‘reset’ demands change to tackle the inequalities, conditions, services, socioeconomic and environmental policies that made people susceptible and vulnerable to COVID-19.
- While economic recovery should not replicate the features of the global economy that are generating pandemic and other crises, for global aspirations to translate into benefit for communities, families, young people and children, an equitable recovery should include significant investment in: (1) universal, public sector, primary health care-oriented health services; (2) redistributive, universal rights-based and life course based social protection; and (3) people, especially in early childhood and in youth, as drivers of change.
- Who designs the ‘reset’ influences the change, and within countries and internationally, opportunities must be provided for meaningful public engagement as a critical driver of an equitable recovery.

## Introduction

One after the other, high-level speakers at the 2020 United Nations General Assembly (UNGASS) on COVID-19 pointed to growing inequalities and stresses to health, social, economic and democratic systems caused by the pandemic, calling for comprehensive, collective interest driven responses.^1^ They called for a sustainable recovery to include: debt relief and international financing; ensuring food security; universal access to vaccines, diagnostics and medicines for COVID-19 as global public goods; military ceasefires to reach populations in conflict areas; and halting ecological determinants of zoonotic pandemics.^1^

These issues will be on international agendas into 2021 and beyond. However, global commitments must translate into benefit for local communities for any recovery to tackle the inequalities and conditions that made society vulnerable to COVID-19, particularly for those experiencing its worst impact.

In this commentary, we examine how COVID-19 has impacted on family and child health and well-being (FCHW) and the implications for a bottom-up recovery. We propose significant investment in universal, public sector, community-driven health and social protection systems to connect measures called for globally with those needed to ensure equitable recovery within communities.

## Pandemic impacts on families, adolescents and children

While the consequences of COVID-19 have varied across countries, women, children, adolescents and marginalised groups, including people with disabilities, have consistently experienced the worst impact.^2 3^

Global progress in achieving the Sustainable Development Goals for women’s, children’s and adolescents’ health already lagged by 20% before the pandemic.^2^ COVID-19 significantly worsened this situation. The closures of health services, including reproductive health services, health worker redeployment and barriers to movement from lockdowns reduced access to care. This led to declining coverage of contraceptives, post-abortion care, maternal health services and immunisation, with 13.5 million children left unprotected from vaccine preventable diseases.^2^ School closures led to 370 million children missing out on learning, school meals and social interaction, affecting child and adolescent mental health, and increasing risks of sexual abuse, gender-based violence and adolescent pregnancy.^2 4^ Social determinants such as poor quality diets, poorly ventilated, overcrowded housing and transport raised susceptibility to and severity of COVID-19.^2 3 5 6^

The responses to COVID-19 have led to their own negative impacts. Lockdowns, service and supply chain disruptions have undermined employment, incomes and increased care burdens, particularly for women. Isolation, uncertainty, coercive measures and disrupted peer relations during lockdowns have triggered fear, stress, stigma and increased gender-based violence and mental disorders.^2 5^ In Australia, adolescents, particularly girls, experienced significant increases in depression and anxiety,^7^ while in Kenya, domestic violence, female genital mutilation and adolescent pregnancy were reported to rise, but also to be under-reported due to barriers to service uptake.^4^ Public health messaging discouraging use of emergency services in Ireland led to decreased paediatric emergency consultations, delaying diagnosis and treatment.^8^ In South Africa, barriers undermined coverage of birth registration, health, education, home visiting and other essential services for children, as well as access to contraceptives and medicines for HIV and chronic conditions.^9^

Years of underinvestment left public health and social systems poorly prepared and overstretched in many countries, especially at community level,^10^ contrasting with the reduced risk and vulnerability in countries with strong community-based primary care, as in Cuba.^11^ In Norway, almost universal, affordable internet access enabled home schooling and remote work. In contrast, barriers to affordable digital access in many low-income countries and communities undermined information sharing, schooling, livelihoods and social participation.^12^ Despite governments implementing over 1400 social protection measures since the pandemic outbreak, the UN observed these to be insufficient, temporary and underfunded, with many gaps in coverage.^10^ In the months of the pandemic, such systemic inequality has increased general and child poverty, while the wealthiest three people in the world have increased their wealth by US$38.5 billion.^10 13^

The pandemic has highlighted the fallacy that economic growth will lead to improved health. Underfunded public services and a reliance on markets, many of which did not work during the pandemic, left many communities exposed. Despite having among the highest levels of economic growth in Latin America, Peru has had the highest levels of mortality from the pandemic in the region, attributed to high levels of social inequality, underinvestment in health and social protection systems and an overfocus on hospital-based intensive care, rather than community and primary care services.^6^

These outcomes signal a rising ‘health and social debt’ in communities, the true scale and long-term consequences of which are as yet unknown, especially for the most marginalised in society.

## A demand and opportunity to transform

With risk and vulnerability to COVID-19 associated with inequity and underinvestment, rather than ‘getting back to normal’, these deficits call for a transformation.

The pandemic has led to recognition at highest political levels that recovery and future preparedness demand investment in public sector health systems and social conditions, with equity, including gender equity, central to their design.^1 10^ Pandemic responses have raised a new lens on the role and performance of the state versus the market in key areas of development and social protection and have demonstrated the benefit from solidarity-driven community responses, supported by local state systems.^14^

A call for a ‘reset’ must be for a *different* way, rather than business as usual. There is an opportunity for change, but also concerns over whose interests will drive the reset. In many countries, social distrust has grown during the pandemic around the fairness, interests, competency, transparency and public accountability of systems and around corruption or bias in use of public funds.^5 15^ Health workers have protested over poor protection and working conditions, and youth and social movements have demonstrated over economic and social choices that have made them vulnerable.^16^

The deficits described earlier point to some critical dimensions of this ‘reset’, if global pronouncements and recovery measures are to equitably benefit families, young people and children.

*First, invest in universal, primary health care (PHC) oriented public health services*. The welcome attention being given to health systems needs to be backed by access to essential health technologies as global public goods and to increased funding. Priority should be given to comprehensive public health and PHC services that provide the best, most equitable and cost-effective first contact for surveillance, prevention, outreach, care and referral for pandemic responses and for addressing the many other areas of growing health need.^5 17^

*Second, establish or strengthen redistributive, universal rights-based and life-course based social protection*. Economic recovery from COVID-19 should not replicate the same features of the global economy that are generating pandemic and other crises. The insecurity driving susceptibility to COVID-19 and widening inequality in its impact suggest that the success of a ‘recovery’ should be assessed in terms of how inclusive, equitable, sustainable it is and how protective of ecosystems it is. One feature of this is in linking socioeconomic measures, services and schemes to establish a more coherent, rights-based and accessible social protection system that addresses gender equity and the different dimensions of vulnerability across the life course.^10^

*Third, recognise and invest in people, especially young people, as drivers of the recovery*. In a global pandemic, it is easy to regard people as objects of action, not actors. This is, for example, often the case in relation to children. However, investment in child well-being is a critical determinant of equity and of a country’s future, and young people can be agents in their own lives, as exemplified in the youth-driven global movement on climate change.^18–20^ Early childhood is the most critical time for development of cognitive, emotional and behavioural capacities that persist throughout life. Deprivation such as that experienced during the pandemic can harm children’s development, but enriching environments can also support recovery.^21^ This calls for holistic, integrated rights-based and culturally appropriate approaches, providing public information and with multidisciplinary, community-based teams delivering the intersectoral investments and public services needed for children to grow and thrive, with meaningful support for parents, families and communities.^20^

## Conclusions: drivers of change

The recovery will generate a noisy debate with competing interests. Before the pandemic, we found that policy attention to FCHW depended on concerted demand across social, political, technical and service actors, engaging those directly affected and promoting solidarity, innovation and shared learning.^19^ Socioeconomic change and service improvements were given impetus by bringing the rights, ideas, voice and agency of those affected into decision making, such as when: citizens and frontline personnel actively shape PHC services; youth and children participate in decisions on services and conditions that affect them; or female workers codesign workplace and social protection systems.^18 19^ Who sits at the table to design the ‘reset’ shapes the change. Within countries and internationally, opportunities must be provided for meaningful public engagement as a critical driver of an equitable ‘reset’.

High-level speakers at the 2020 UNGASS on COVID-19 expressed welcome ambitions for a recovery that would restructure the international finance architecture, protect the planet and use multilateral mechanisms for collective interests and security. However, it should not stop there. Democracy, collective security and socioeconomic equity *between* countries also need to apply *within* countries. Participatory, integrated approaches and investments in areas such as universal, PHC-oriented public health services; redistributive, universal rights-based and life-course based social protection, and investment in children, youth, parents, families and communities are essential to connect the values, changes and resources called for internationally with the substantive changes needed for an equitable recovery within society.
